# Sexual behavior of university students in the context of the COVID-19 pandemic: a mixed-methods study*

**DOI:** 10.1590/1980-220X-REEUSP-2023-0116en

**Published:** 2024-02-02

**Authors:** Maria Hellena Ferreira Brasil, Anna Cláudia Freire de Araújo Patrício, Wynne Pereira Nogueira, Maria Eliane Moreira Freire, Elucir Gir, Ana Cristina de Oliveira e Silva

**Affiliations:** 1 Universidade Federal da Paraíba, Programa de Pós-Graduação em Enfermagem, João Pessoa, PB, Brazil.; 2 Universidade Federal da Paraíba, Escola Técnica de Saúde, João Pessoa, PB, Brazil.; 3 Universidade de São Paulo, Escola de Enfermagem de Ribeirão Preto, Ribeirão Preto, SP, Brazil.

**Keywords:** Sexual Behavior, Students, Sexually Transmitted Diseases, COVID-19, Epidemiology, Conducta Sexual, Estudiantes, Enfermedades de Transmisión Sexual, COVID-19, Epidemiología, Comportamento Sexual, Estudantes, Infecções Sexualmente Transmissíveis, COVID-19, Epidemiologia

## Abstract

**Objective:**

To analyze the sexual behaviors of university students during the COVID-19 pandemic.

**Method:**

Mixed study, carried out on four campuses of a public university in Paraíba, Brazil, between March 2021 and April 2022. The research followed ethical precepts.

**Results:**

404 university students were included, with an average age of 23.7 years, predominantly female, brown and single. The prevalence of self-reported sexually transmitted infections was 7.9%. Male students were more likely to engage in risky sexual behavior. Multiple logistic regression indicated that university students aged 25 or over who had engaged in casual sex in the last 12 months and had received or paid for sex were more likely to have sexually transmitted infections. The content analysis showed that social isolation was reflected in reduced consumption of alcohol and other substances, reduced sexual practices, increased use of social networks, as well as low adherence to condoms.

**Conclusion:**

Physical distancing has an impact on the sexual behavior of university students, as well as on the consumption of alcohol and other substances.

## RESUMO

**Objetivo:**

Analisar os comportamentos sexuais de estudantes universitários durante a pandemia da COVID-19.

**Método:**

Estudo misto, realizado em quatro campus de uma universidade pública na Paraíba, Brasil, entre março de 2021 e abril de 2022. A pesquisa seguiu os preceitos éticos.

**Resultados:**

Foram incluídos 404 universitários, com idade média de 23,7 anos de idade, com predomínio do sexo feminino, pardo e solteiro. A prevalência de infecções sexualmente transmissíveis por autorrelato foi de 7,9%. Estudantes do sexo masculino apresentaram com maior frequência a prática de comportamentos sexuais de risco. A regressão logística múltipla indicou que universitários com idade igual ou superior a 25 anos, que praticaram relação sexual casual nos últimos 12 meses e já receberam ou pagaram em troca de relação sexual apresentaram maiores chances de incidência de infecções sexualmente transmissíveis. A análise de conteúdo evidenciou que o isolamento social refletiu na diminuição do consumo de álcool e outras substâncias, redução de práticas sexuais, aumento do uso de redes sociais, assim como na baixa adesão ao preservativo.

**Conclusão:**

O distanciamento físico repercutiu no comportamento sexual de estudantes universitários, assim como no consumo de álcool e outras substâncias.

## RESUMEN

**Objetivo:**

Analizar el comportamiento sexual de estudiantes universitarios durante la pandemia de COVID-19.

**Método:**

Estudio de método mixto realizado en cuatro campus de una universidad pública de Paraíba, Brasil, entre marzo de 2021 y abril de 2022. La investigación siguió preceptos éticos.

**Resultados:**

Fueron incluidos 404 estudiantes universitarios, con edad media de 23,7 años, predominantemente del sexo femenino, castaños y solteros. La prevalencia de infecciones de transmisión sexual autodeclaradas fue del 7,9%. Los estudiantes varones eran más propensos a tener conductas sexuales de riesgo. La regresión logística múltiple indicó que los estudiantes universitarios de 25 años o más, que habían mantenido relaciones sexuales ocasionales en los últimos 12 meses y que habían recibido o pagado por mantener relaciones sexuales tenían más probabilidades de padecer infecciones de transmisión sexual. El análisis de contenido mostró que el aislamiento social se reflejaba en una reducción del consumo de alcohol y otras sustancias, una reducción de las prácticas sexuales, un aumento del uso de redes sociales, así como una baja adherencia al preservativo.

**Conclusión:**

El distanciamiento físico influye en el comportamiento sexual de los universitarios, así como en el consumo de alcohol y otras sustancias.

## INTRODUCTION

Sexually Transmitted Infections (STIs) are considered a relevant public health issue. Around 360 million cases of STIs are diagnosed annually worldwide, with an incidence of 10 to 12 million in Brazil. It is estimated that 25% of cases occur in the young population, up to the age of 25^(1)^.

Data published by the Ministry of Health, Brazil, for the year 2020, in relation to HIV/AIDS, show an increase in the detection rate of the disease among young people aged 15 to 24, with 33.2 cases/100,000 inhabitants, compared to 27.2 cases/100,000 inhabitants in the year 2010. The highest incidence was found among people aged between 25 and 29, with 43.2 cases/100,000 inhabitants^(2)^.

Young people are considered to be the part of the population most exposed to STIs, such as HIV/AIDS and acquired syphilis. This is associated with economic, social and individual factors. Among the individual factors are the physical, biological and hormonal changes brought about by adolescence, which have a direct impact on sexual initiation and the possibility of multiple partners^(3,4)^.

In addition to youth, entering university is considered a time of physical, psychological, social and behavioral changes for individuals. The university environment promotes opportunities for sexual experimentation and the practice of risky sexual behavior (RSB), since it is often the first time that young people are experiencing freedom from their parents and guardians^(5)^.

University students are considered a population segment at risk of STIs, as they tend to have a distorted sense of vulnerability to these infections. Among the aspects related to the vulnerability of university students to STIs, in addition to the practice of RSB, are insufficient knowledge about these infections, unfavorable social status and weaknesses in public policies aimed at this population^(6)^.

RSB is defined as the practice of actions that can lead to the risk of contracting STIs, unwanted pregnancies, unsafe abortions and psychosocial dysfunctions. Among the main CSR in university students, the following stand out: early sex maturity, multiple partners, recruiting partners through dating apps and resistance to condom use. In addition, the use of alcohol and other licit and illicit substances is associated with an increased incidence of RSBs in this group^(7,8)^.

In March 2020, the World Health Organization (WHO) declared a pandemic due to the disease caused by the new coronavirus, widely known as COVID-19. Due to its form of transmission, mostly via the respiratory route, the initial guidelines were to implement isolation and physical distancing strategies, in order to contain the viral spread and avoid the collapse of health systems^(9)^.

Physical distancing, a non-pharmacological measure to prevent COVID-19, especially in the first year of the pandemic, may have affected individuals’ sexual behavior. There is still a lack of studies revealing whether this measure has influenced a reduction in casual sex or led to an increase in SRB in university students^(10)^.

The COVID-19 pandemic has had an impact on the daily lives of young people, especially those who attend university. The context of uncertainty provided by the global health crisis has directly influenced the emergence of feelings such as anguish, loneliness, anxiety, tension and decreased quality of life. Among the strategies used by students to reduce their mental suffering are sexual relations and the use of alcohol and other substances^(11,12)^.

It is therefore possible to understand that young people are the section of the population with the highest incidence of STIs and that going to university often represents opportunities for practicing RSB. In addition, the scenario caused by the pandemic has proved to be conducive to behavioral changes, especially in university students, since studies show that non-pharmacological measures to prevent infection have had a negative impact on the routine of these individuals.

This article highlights the relationship between non-pharmacological measures to prevent COVID-19 and the sexual behavior of university students, given that the pandemic has had an impact on the population’s sexual health. The aim of this study was to analyze the sexual behavior of university students during the COVID-19 pandemic.

## METHOD

### Study Design

This is a mixed-method study with a sequential explanatory design (DEXPLIS), derived from a master’s thesis. DEXPLIS is considered a valuable strategy, as it collects both quantitative and qualitative data, promoting a more intense understanding of the phenomenon studied. The quantitative data should be collected and analyzed first, followed by the qualitative data^(13)^.

### Study Site

The research was carried out on four campuses of a public university in the state of Paraíba, Brazil. The campuses were located in different cities, making it possible to analyze the reality of different regions of the state.

### Selection Criteria

University students who met the following inclusion criteria took part in the study: age 18 or over, enrolled in an undergraduate course at the institution before the start of the COVID-19 pandemic (March 2020) and who were compulsorily studying subjects in 2020. Students from courses in all major areas of knowledge were included. Technical and postgraduate students were excluded.

### Sample Definition

For the sample calculation, we opted for stratified sampling, in which we considered the number of students at each of the institution’s campuses. A population of 27,757 students was obtained from the four campuses. After calculating the sample size obtained by the stratification procedure, considering a simple random sampling plan in each group, the calculated sample size, with a loss forecast of around 20%, was 403 students, giving a total of 404 interviewees. As this is a mixed-method study, participants were selected to compose the sample to obtain qualitative data by convenience, applying the data saturation technique to make up the final sample, totaling 21 university students. The qualitative data collection was considered saturated when the speeches were repeated and no new elements were found^(14)^.

### Data Collection

Data collection took place between March 2021 and April 2022, in two phases. To operationalize the collection of the first phase (quantitative data), a team of previously trained collaborators, composed by undergraduate and postgraduate nursing students, was used. The second phase (semi-structured interviews) was carried out privately by the researcher. Quantitative data was collected using a structured questionnaire made up of three sections. Section I contained questions about sociodemographic characteristics; section II addressed sexual behavior (age at onset of sexual activity, number of partners in the last year, types of sexual practices, frequency of condom use, recruitment of partners through social networks, as well as previous STIs and their respective signs and symptoms) and section III asked about the use of licit and illicit drugs. Participants in the first phase were invited to take part in the qualitative phase. The students who agreed to take part were contacted afterwards to carry out the semi-structured interview, characterizing the second phase, with the aim of promoting a deeper understanding of the phenomenon studied, assessing the subjectivity present in the statements. The interview contained five questions about students’ sexual behavior during the pandemic, the use of social networks and/or dating apps to recruit partners, the impact of physical distancing on affective and sexual relationships, and the use of alcohol, tobacco and other substances during the pandemic. The average duration was 20 minutes. The interviews were recorded on a private cell phone and then transcribed.

### Data Analysis and Processing

The quantitative data was tabulated in an Excel^®^ spreadsheet (Microsoft^®^ 365, version 2019) and then imported into the Statistical Package for Social Sciences, version 26.0 for Windows^®^. The analysis was carried out using descriptive and inferential statistics. The prevalence of self-reported STIs was calculated with a 95% confidence interval (95%CI). To investigate the risk factors and behaviors associated with the prevalence of infections, a bivariate analysis was first carried out using the Chi-square test.

The variables that were statistically significant were included in the binary logistic regression model, generating odds ratios (OR) with 95% CI to infer whether the independent variables (sociodemographic or sexual behavior) were a risk or protective factor for the occurrence of the reported STI (dependent variable). It should be noted that in order to calculate the OR, the variables were previously dichotomized. The degree of association between the factors was inferred by the OR value: above 1 (risk factor) or below 1 (protective factor)^(15)^.

Variables with a p < 0.25 were simultaneously included in the multiple logistic regression model using the stepwise method. In the final model, variables with a statistically significant association of p < 0.05 were considered.

The semi-structured interviews were listened to and transcribed by the researcher in a text program and imported into NVivo 12^®^ software, free version, enabling thematic categorical content analysis^(13)^. An inductive approach was used to categorize the results, i.e. the categories were defined after the data had been analyzed^(16)^.

### Ethical Aspects of the Research

This study followed the recommendations set out in Resolution 466/2012 of the National Health Council (CNS/MS/BRASIL) regarding scientific research involving human beings. To this end, the project was cleared by the Research Ethics Committee of the Health Sciences Center of the Federal University of Paraíba, according to opinion No. 4.309.767/2020. It should be noted that the university students who took part in the study were asked to read and sign the Free and Informed Consent Form (FICF), in two copies, before the data collection began. In order to preserve the anonymity of the statements made by the participants in the second phase of the study, a code was used according to the order in which the interviews were conducted, plus age and gender information (Participant 1, Participant 2… Participant 21, age and gender).

### RESULTS

A total of 404 (100%) university students from the state of Paraíba took part in the study. The majority were female (biological) 234 (57.9%), aged between 18 and 24, 291 (72.0%), brown 174 (43.1%), single 352 (87.1%), religious 219 (54.2%), with a monthly family income of two minimum wages or less 241 (59.7%), not living in a university residence 294 (72.8%) and receiving some student assistance 271 (67.1%).

The prevalence of self-reported STIs among the participants was 7.9% (95%CI: 7.1–8.6). Among these, syphilis was the most frequent, 11 (2.7%), followed by HPV, 10 (2.5%). Of the students who self-reported the presence of an STI, the most frequent clinical sign was the presence of sores and warts in the genital area 13 (40.6%).

Bivariate analysis of the association between sociodemographic characteristics (independent variables) and the prevalence of STIs (dependent variable) showed that male students (p < 0.001), those aged 25 or over (p = 0.001) and those living in a university residence (p = 0.009) were more likely to have STIs.

As for the association between risk behaviors and biological sex (male and female), it was observed that male students were more likely to have had sexual intercourse before the age of 15 (p = 0.006), sexual intercourse with a person of the same sex (p = 0.001), casual sexual intercourse in the last 12 months (p < 0.001), sexual intercourse with a sex worker (p < 0.001) and to have paid or received money in exchange for sexual intercourse (p < 0.001).

In the association between the variables of the main risk behaviors associated with the presence of STIs, it was observed that the variables “age at first sexual intercourse” (p = 0.013), “sexual intercourse with a person of the same sex” (p < 0.001), “casual sexual intercourse in the last 12 months” (p < 0.001), “sexual intercourse with a sex worker” (p = 0.030), “received money or paid in exchange for sexual intercourse” (p < 0.001), “sexual intercourse with a partner met on a cell phone” (p < 0.001) and smoking (p < 0.001) were statistically significant (Table 1).

**Table 1:** Association between risky sexual behavior and the presence of STIs in university students – PB, Brazil, 2022 (n = 404).

Variables	Presence of STI		p-value
	Yes (n = 32) n (%)	No (n = 372) n (%)	
**Age of first sexual intercourse**			0.013*
≤15 years	12 (14.5)	71 (85.5)	
>15 years	20 (6.2)	301 (93.8)	
**Sexual intercourse with** **person of same sex**			<0.001*
Yes	22 (13.8)	138 (86.2)	
No	10 (4.1)	234 (95.9)	
**Frequency of condom use in** **the last 12 months**			0.354
Always/sometimes	27 (10.0)	243 (90.0)	
Never	3 (5.9)	48 (94.1)	
**Casual sexual intercourse in** **the last 12 months**			<0.001*
Yes	28 (13.0)	188 (87.0)	
No	4 (2.1)	184 (97.9)	
**Sexual relationship with sex** **worker**			0.030*
Yes	4 (21.1)	15 (78.9)	
No	28 (7.3)	357 (92.7)	
**Ever received money or paid** **in exchange for sex**			<0.001*
Yes	9 (34.6)	17 (65.4)	
No	23 (6.1)	355 (93.9)	
**Ever had sex with a sexual** **partner you met on your cell** **phone**			<0.001*
Yes	25 (13.3)	163 (86.7)	
No	7 (3.2)	209 (96.8)	
**Smoking**			<0.001*
Yes	15 (18.5)	66 (81.5)	
No	17 (5.3)	306 (94.7)	
**Ever used illicit drugs**			0.157
Yes	20 (9.8)	184 (90.2)	
No	12 (6.0)	188 (94.0)	

* Statistically significant association, p < 0,05.

The final logistic regression model showed that university students aged 25 or over, who had had casual sex in the last 12 months and who had received money or paid in exchange for sex were more likely to have STIs (Table 2).

**Table 2:** Multiple logistic regression analysis for the presence of STIs in university students – PB, Brazil, 2022 (n = 404).

Variables	Adjusted odds ratio	CI 95%*	p-value
Age			
18–24 years	1	1.18–5.75	0.018**
≥25 years	2.60		
Casual sex relationship in the last 12 months			
Yes	3.49	1.37–10.7	0.029**
No	1		
Ever received money or paid in exchange for sex			
Yes	3.28	1.19–9.05	0.021**
No	1		

* CI95% confidence interval 95%.

** Statistically significant association, p < 0.05.

Phase 2 of the study explored the influence of the pandemic on the sexual behavior of university students.

In this phase, 21 (100%) students took part, the majority female 13 (61.9%), aged between 18 and 24 years 14 (66.7%), brown 11 (52.4%), single 20 (95.2%), with religion 12 (57.2%), monthly family income of more than two minimum wages 15 (71.4%), who do not live in the university residence 15 (71.4%) and who receive some student assistance 15 (71.4%).

After analysis using NVivo^®^ software, the following categories emerged: 1) “Influence of the social environment on the use of alcohol, tobacco and other substances”; 2) “Reduction in sexual practices during the pandemic”; and 3) “Risky sexual behavior during the pandemic”, with two subcategories: 3.1) Increased use of social networks and/or dating apps in the last 12 months and 3.2) Low adherence to condom use during the pandemic.

### Influence of the Social Environment on the Use of Alcohol, Tobacco and Other Substances

Participants reported that adherence to non-pharmacological measures to prevent COVID-19, especially physical distancing, helped to reduce the use of alcohol, tobacco and other substances.

*Speaking personally, my alcohol consumption has decreased during the pandemic, since my consumption was basically at social events and in public places, going out with friends, these things, since this has decreased drastically, my consumption has also decreased drastically (Participant 4, 23 years old, male)*.

*It has decreased. Because normally I don’t use much, I use it more socially, and since there were no crowds or anything, it decreased a lot (Participant 12, 24 years old, male).*

*In my case, I’ve never had a problem with any substance... I drink alcohol when I want to, it’s not a desire to “wow, I need to drink alcohol”, so as I had nowhere to go out, there were no parties, barbecues, these things because of the pandemic... so there was a big decrease in alcohol, I only drank when I was going out, at home I don’t drink alcohol (Participant 17, 22 years old, female).*

It can be inferred from the speeches that the social environment influences the practice of risky behaviors. The consumption of alcohol and other substances tends to be lower in family environments, as reported by participants of both genders.

### Reduction in Sexual Practices During the Pandemic Period

Still in the context of changing behaviors due to the COVID-19 pandemic, students reported a reduction in sexual practices.

*Yes, the pandemic has affected the sexual context, because I ended a relationship, and because of that my sexual relations kind of stopped. The logistics of the relationship became unfeasible due to the pandemic (Participant 19, 20 years old, female).*

*(...) there was a big decrease in sexual behavior, which was almost nil, at least in the first year of the pandemic, precisely for my own well-being, it wasn’t a priority at any time (Participant 4, 23 years old, male).*

*As for sexual relations, these were also negatively affected during the pandemic, also due to the distancing, I tried to avoid relationships with other people (Participant 20, 23 years old, male).*

Physical distancing was a COVID-19 mitigation measure that had an impact on reducing sexual practices, both in romantic and casual relationships.

### Risky Sexual Behavior During the Pandemic

Although the majority of students reported a reduction in sexual practices, it was still possible to see reports of RSB, as presented in the following subcategories:

### Increased Use of Social Networks and/or Dating Apps in the Last 12 Months

The university students’ speeches showed that there had been an increase in the search for sexual partners mediated by the use of social networks and/or dating apps.

*(...) I started looking for, like... apps, other possibilities, and then I think my perspectives changed (Participant 2, 20 years old, female).*

*(...) My girlfriend and I ended up getting into something more monotonous, we went in search of apps and threesomes, so that affected me a lot. It happened because the relationship was a bit dull, and we wanted to do something different (Participant 1, 23, female).*

*(...) we felt more isolated, so there was more demand for apps, for a certain period (Participant 13, 31 years old, male).*

Considering that the pandemic has reduced opportunities for social gatherings, many young people have turned to dating apps in order to recruit partners for sex, whether in person or online.

### Low Adherence to Condom Use During the Pandemic

With regard to this subcategory, it was possible to infer that the majority of participants did not use condoms properly.

*(...) I’ve had condoms tear during sex and the person didn’t tell me, I’ve had drunk sex without a condom (Participant 2, 20 years old, female).*

*I had a partner and we did use condoms, because my menstrual cycle is very irregular and we did it with the intention of not having an unwanted pregnancy. So... the question of diseases, as it’s a single partner, and I believe he’s not cheating on me, we do it to prevent pregnancy (Participant 5, 24 years old, female).*

*In the last three months I’ve only had one partner... I didn’t use it initially during sexual intercourse, because we already had the intimacy of having sex without a condom, but always avoiding getting pregnant, these things, we used condoms at the end of sexual intercourse, but I had sex without a condom (Participant 12, 31 years old, male).*

*It’s been more than a year, more or less, almost 2 years, it’s the same sexual partner and we don’t usually use condoms, it’s kind of become a custom of the couple, because they think it’s uncomfortable or they don’t feel as much pleasure as without a condom (Participant 3, 28 years old, female).*

Most of the participants reported using condoms inappropriately, which leads to a risk of STI transmission. Among those who reported using condoms, there was a greater concern with contraception than with preventing these infections.

## DISCUSSION

This study presents data on the sexual behavior of university students in the context of the pandemic, considering the use of alcohol and other substances, strategies for recruiting partners and adherence to non-pharmacological measures to prevent COVID-19. It is important to note that during the data collection period there was a high incidence of cases of the aforementioned infection. The second phase of the study showed that compliance with measures to mitigate viral transmission, especially physical distancing, led to a reduction in the use of alcohol and other substances, as well as a reduction in sexual practices in most of the participants. However, there were still reports of RSB.

In the sociodemographic characterization of the participants, the sample consisted mostly of women, single and under the age of 25, as in studies with university students in Italy and Africa^(11,12)^.

With regard to the higher prevalence of STIs in male students, a study in Asia showed that men tend to have a more active sex life than women, as well as higher rates of alcohol and other substance use before sex, behaviors that are considered to be at risk of STI transmission. In addition, they were less knowledgeable about sexual health and infection prevention when compared to women, which makes them more vulnerable to contracting these infections^(3)^.

Added to this context, a study in Uganda found that male university students were twice as likely to practice RSB when compared to females^(17)^. In addition, a study in southern Brazil found that men tend to start having sex earlier than women, which is associated with practicing RSB in adulthood^(7)^.

A study in Tanzania found that those who started sexual activity at an earlier age (≤16 years) were more likely to report STI symptoms than those who started after the age of 17, which is associated with a low level of knowledge about infections among the younger population^(18)^.

A study carried out in Africa found that university students over the age of 24 are around 2.12 times more likely to engage in RSB when compared to those under this age, a result that corroborates the findings of the present study. This may be related to the increase in sexual desire when young people become physically and physiologically more mature^(6)^.

Living in a university residence is often the only option for economically vulnerable university students to continue their studies. The change from the family environment to living with other students can have an impact on the psychological and social contexts of these individuals, awakening a sense of freedom, independence and autonomy^(19)^. It is believed that these feelings are associated with the practice of RSB, but there are few studies on this.

As for the association between the variables “same-sex sexual intercourse” and STIs, research shows that despite awareness campaigns about strategies to prevent such infections, the prevalence of STIs among this group remains high. HIV detection rates are higher among men who have sex with men (MSM). This population reveals that low adherence to prevention methods is explained by discomfort in using condoms, low knowledge about the effectiveness of the methods, use of psychoactive substances before intercourse, embarrassment at suggesting use to their partner and casual sex^(5,20)^.

In this study, the increased use of social networks favored the practice of casual sex. A survey of 7,678 Polish university students found that 33% said they practiced casual sex. Such practices are generally associated with RSBs, especially when associated with the use of alcohol and other substances^(21)^.

Regarding the greater chances of STIs among individuals who have paid or received in exchange for sexual intercourse, a study in Ethiopia indicates that this practice tends to pose a high risk of transmitting these infections, given that the practice is often not carried out safely^(22)^. There is a scarcity of studies in the literature dealing with this issue among university students.

Concurring with this research, an investigation with 800 Iranian university students found that frequenting bars and nightclubs, as well as smoking cigarettes, were associated with the practice of RSB. These attitudes were more prevalent among male students^(8)^.

Considering that the use of alcohol and other substances is associated with the practice of RSB, it was observed through the interviews that during the pandemic there was a reduction in the use of such substances, as in studies at Spanish^(23)^ and Swedish^(24)^ universities.

In the meantime, the authors state that the decrease in the consumption of these substances was related to the lockdown, which restricted social gatherings. It was also observed that students tend to consume substances in smaller quantities in the family environment, similar to what was found in this study^(23,24)^.

The impact of the pandemic on love relationships was very present in the university students’ speeches. The literature shows that the stressors caused by the pandemic, such as physical distancing, changes in educational format and unemployment, have had a negative influence on the physical, social and mental health of young people. As such, many of the relationships in which there was no cohabitation were affected, as the distancing led to a reduction in quality time with partners, which, added to the aforementioned stressors, often culminated in conflicts^(25)^.

Regarding the reduction in sexual practices during the pandemic period, a qualitative study in two states in different regions of Brazil found that most of the participants stopped having sex as often as they did as a result of physical distancing. In addition, it was possible to see that there was a feeling of fear of becoming infected and transmitting COVID-19 to loved ones, culminating in high adherence to isolation, especially in the first year of the pandemic^(26)^.

In a study in China, 292 (53.9%) university students who searched for sexual partners on the internet had casual sex. Also in this study, having sex with partners known through social networks implied greater chances of practicing RSB (OR: 4.434; p < 0.001)^(27)^.

Dating apps are often used to promote quick or even immediate sexual encounters, since the search is carried out according to location. Users of these tools tend to engage in more RSB than non-users, such as the ease of having multiple partners, inconsistent condom use, casual sex and increased use of alcohol and other substances during encounters, which can lead to an increase in STI rates in this group^(28,29)^.

Considering condom use, a study in Rio de Janeiro, Brazil, found that 70.37% of university students did not use condoms during their first sexual intercourse, most of which took place between the ages of 15 and 18; in addition, 60.74% said they did not use this preventive method in all sexual relations, a habit that was more common among those in stable unions^(30)^.

The limitations of this study are related to the fact that it only analyzed the reality of public higher education institutions, which would require a study with students from both public and private schools. In addition, as it deals with the context of a single state, it would be important to replicate the study in other states of the country in order to gain a broader understanding of the issue. However, it is believed that the results presented represent the beginning of future studies involving contemporary sexual risk behaviors among university students, such as recruiting partners through social networks. Thus, as implications for professional practices, knowing the reality of these university students implies the condition of creating important subsidies in the strategies for prevention and promotion of the sexual and reproductive health of the young population in the modern world.

## CONCLUSION

This research provides data on the sexual behavior of university students in the context of the COVID-19 pandemic, a topic that is still infrequent in the scientific literature. The mixed method provided a deeper understanding of these behaviors.

The following variables were statistically associated with the presence of STIs: male sex, age 25 or over, living in a university residence, first sexual intercourse ≤15 years, sexual intercourse with a person of the same sex, casual sexual intercourse in the last 12 months, sexual intercourse with a sex worker, receiving or paying for sexual intercourse, sexual intercourse with a partner met by cell phone and smoking. Male (biological) students were more likely to practice RSB.

As for the impact of the pandemic context on sexual behavior, the discourses of students of both (biological) sexes showed that there was a decrease in the consumption of alcohol and other substances, a reduction in sexual practices, an increase in the use of social networks and dating apps to recruit sexual partners and low adherence to condom use. These practices were closely related to non-pharmacological measures to mitigate COVID-19, especially physical distancing.

The results reinforce the need to invest in strategies and public health policies on health promotion and disease prevention aimed at the aforementioned target audience, since it is a group at risk of practicing SRH, considering the age group, the university environment and the context caused by the pandemic. It is essential to carry out health education activities that address sexual and reproductive health in universities, as well as the continuous offer of rapid tests for STI detection, considering that early diagnosis is essential for a good prognosis.
